# Low Hemoglobin-to-Red Cell Distribution Width Ratio Is Associated with Disease Progression and Poor Prognosis in Upper Tract Urothelial Carcinoma

**DOI:** 10.3390/biomedicines9060672

**Published:** 2021-06-11

**Authors:** Yung-Chun Su, Sheng-Chen Wen, Ching-Chia Li, Hsiao-Chun Su, Hung-Lung Ke, Wei-Ming Li, Hsiang-Ying Lee, Chia-Yang Li, Sheau-Fang Yang, Hung-Pin Tu, Wen-Jeng Wu, Hsin-Chih Yeh

**Affiliations:** 1 School of Medicine, College of Medicine, Kaohsiung Medical University, Kaohsiung 80708, Taiwan; joe19960301@gmail.com (Y.-C.S.); u106001135@gap.kmu.edu.tw (H.-C.S.); 2 Department of Urology, Kaohsiung Medical University Hospital, Kaohsiung 80756, Taiwan; carl0815@gmail.com (S.-C.W.); ccli1010@hotmail.com (C.-C.L.); hunglungke@yahoo.com.tw (H.-L.K.); u8401067@yahoo.com.tw (W.-M.L.); ashum1009@hotmail.com (H.-Y.L.); wejewu@kmu.edu.tw (W.-J.W.); 3 Department of Urology, School of Medicine, College of Medicine, Kaohsiung Medical University, Kaohsiung 80708, Taiwan; 4 Graduate Institute of Medicine, College of Medicine, Kaohsiung Medical University, Kaohsiung 80708, Taiwan; chiayangli@kmu.edu.tw; 5 Department of Urology, Ministry of Health and Welfare Pingtung Hospital, Pingtung 90054, Taiwan; 6 Cohort Research Center, Kaohsiung Medical University, Kaohsiung 80708, Taiwan; 7 Department of Urology, Kaohsiung Municipal Ta-Tung Hospital, Kaohsiung 80145, Taiwan; 8 Graduate Institute of Clinical Medicine, College of Medicine, Kaohsiung Medical University, Kaohsiung 80708, Taiwan; 9 Department of Pathology, Kaohsiung Medical University Hospital, Kaohsiung 80756, Taiwan; sfyang@kmu.edu.tw; 10 Department of Pathology, School of Medicine, College of Medicine, Kaohsiung Medical University, Kaohsiung 80708, Taiwan; 11 Department of Public Health and Environmental Medicine, School of Medicine, College of Medicine, Kaohsiung Medical University, Kaohsiung 80708, Taiwan; p915013@kmu.edu.tw

**Keywords:** upper tract urothelial carcinoma, white blood cell count, hemoglobin, red blood cell distribution width, progression, prognosis

## Abstract

The importance of blood cell markers in patients with malignant tumors has been studied, but there are few studies on the prognostic value of hemoglobin-to-red cell distribution width ratio (HRR) in cancer. This is the first study to investigate the effect of preoperative HRR on patients with upper tract urothelial carcinoma (UTUC). Our retrospective cohort study included 730 UTUC patients who underwent nephroureterectomy from 2000 to 2019. Clinicopathological parameters were compared according to HRR levels, and the relationship between blood cell markers (HRR, white blood cell [WBC] count, platelet count) and prognosis was evaluated using Kaplan–Meier method and Cox regression model. We found that patients with HRR ≤ 1.05 tended to have worse renal function, higher pathological stages, and more high-grade tumors. In univariate analysis, HRR ≤ 1.05, WBC > 8.65 × 10^3^ cells/μL and platelets >309 × 10^3^ cells/μL were associated with poor progression-free survival (PFS), cancer-specific survival (CSS), and overall survival (OS). Multivariate analysis demonstrated that HRR ≤ 1.05 and WBC > 8.65 × 10^3^ cells/μL were independent prognostic factors for predicting deterioration of PFS, CSS, and OS. In conclusion, HRR and WBC are easy to obtain in clinical practice and are useful indicators to provide prognostic information before surgery for UTUC.

## 1. Introduction

Although upper tract urothelial carcinoma (UTUC) is a rare cancer that accounts for no more than 10% of urothelial carcinomas [1], its incidence is gradually increasing [2]. In particular, according to the 2018 Annual Report of the Taiwan Cancer Registry, UTUC comprises up to 40% of all urothelial carcinomas, which is unique compared to other countries. Unlike Western populations [3], the gender distribution of UTUC patients in Taiwan is dominated by women. A multicenter cohort study of 1436 patients from 15 institutions in Taiwan showed that 58% were female [4]. Currently, radical nephroureterectomy with bladder cuff excision remains the gold standard for the treatment of high-risk UTUC [5]. Nevertheless, even after radical surgery, 28% of patients still relapse in a median follow-up of about 3 years, and 23% can die of the disease [6]. In addition, while there are several important prognostic factors based on the final pathology of surgical specimens, there is a lack of reliable preoperative biomarkers.

Therefore, researchers around the world have been searching for useful prognostic indicators to identify suitable candidates for adjunct therapy. Blood cell markers, including white blood cell (WBC) count, platelet count, hemoglobin and red blood cell distribution width (RDW), are routinely available before surgery, and their significance in UTUC has been widely discussed [7,8,9,10,11,12,13,14,15,16,17,18,19,20]. Both WBC count and platelet count are closely related to cancer outcomes. It has been reported that cancer-associated bone marrow proliferation leads to increased WBC count and immunosuppression, and platelets can help form a permissive microenvironment and expand metastasis routes [21,22].

Hemoglobin and RDW are markers derived from red blood cells. It is believed that they can reflect the oxidative stress [23,24] and inflammation [25,26] and, therefore, are correlated with the prognosis of cancer [9,10,11,12,13,14,15,16,17,18,19,20]. The hemoglobin-to-RDW ratio (HRR) is a novel prognostic marker, and Sun et al. conducted the first study linking HRR and cancer [27]. They discovered that in patients with esophageal cancer, HRR < 0.989 was a significant factor for poor overall survival (OS) with a hazard ratio (HR) of 1.416 by multivariate analysis. The prognostic value of HRR in UTUC has not been examined. In this study, we aim to investigate the significance of HRR and other blood cell markers in UTUC patients treated with nephroureterectomy.

## 2. Materials and Methods

This retrospective cohort study was approved by the institutional review board of Kaohsiung Medical University Hospital (KMUH-IRB-20180214), which waived the need for formal informed consent for this type of study. All procedures performed in research involving human participants were in compliance with the ethical standards of the institutional research committee and with the 1964 Helsinki declaration. Patients who lacked complete clinicopathological or laboratory data were excluded. Specifically, 182, 118, 3, and 2 cases were excluded due to lack of complete blood count, RDW, pathological stage, and tumor focality, respectively. A total of 730 Asian patients who received nephroureterectomy for UTUC from 2000 to 2019 were included for analysis. UTUC were diagnosed through ultrasound, urinary cytology, intravenous urography, computed tomography, magnetic resonance imaging, or ureteroscopy. Radical nephroureterectomy was performed openly or laparoscopically according to the patient’s choice and the surgeon’s preference.

Patient information was obtained from medical charts and pathology reports, including age, gender, smoking, history of bladder cancer, tumor location, surgery approach, pathological T and N stages, tumor grade, focality and laboratory data. Tumors were staged according to the 2010 TNM system, the AJCC (American Joint Committee on Cancer) Cancer Staging Manual (7th edition). All patients underwent routine laboratory tests before surgery, including renal function and complete blood count. The stage of chronic kidney disease was determined by the estimated glomerular filtration rate, which was calculated using the MDRD (Modification of Diet in Renal Disease) study equation: 186 × (serum creatinine)^−1.154^ × (Age)^−0.203^ × (0.742 if female) [28]. The details of blood cell count, such as WBC count, platelet count, hemoglobin, and RDW, were collected for analysis. HRR was defined as hemoglobin (g/dL) divided by RDW (%) [27]. We analyzed all possible values within the central 80% distribution of the continuous variable to acquire the cutoffs that best distinguished the survival rate of patients. Progression-free survival (PFS), cancer-specific survival (CSS), and OS were the time from the date of surgery to each endpoint, namely disease progression, cancer-specific death, all-cause mortality, or last visit.

Following the institutional guideline, urine cytology and cystoscopy were performed every three months for the first two years after surgery, every six months for the next two years, and once a year thereafter. Patients regularly received chest X-ray, abdominal ultrasound, and computed tomography during the follow-up. Progression was defined as recurrence at the local surgical site, lymph node involvement, and distant metastasis. Tumors that recurred in the contralateral upper urinary tract or bladder were not considered relapses.

In this study, all analyses were performed with SPSS statistical software version 22 (IBM, Armonk, New York, NY, USA). Continuous variables were presented as mean ± standard deviation, and categorical variables were presented as counts and percentages. Pearson chi-square test and Student’s *t* test were used to compare categorical variables and continuous variables, respectively. For all statistics, *p* < 0.05 was considered significant. Survival rates were analyzed by the Kaplan–Meier method, and survival curves were compared with the log-rank test. The Cox regression model was used to determine the significant predictors of PFS, CSS, and OS in univariate analysis, and these significant parameters were incorporated into multivariate analysis to identify independent prognostic factors. Specifically, we did not include hemoglobin or RDW in the multivariate model to prevent overadjustment and collinearity to HRR.

## 3. Results

### 3.1. Patient Characteristics

The study recruited 730 patients, including 407 females and 323 males. The clinical and pathological characteristics of the patients are summarized in Table 1. The mean age of this population was 67.36 ± 10.48 years. A total of 21% of patients smoked, and a history of bladder cancer was noticed in 111 patients (15.2%). Of all 730 cases, 319 (43.7%) had tumors in the renal pelvis, 270 (37.0%) had tumors in the ureter, and the remaining cases (19.3%) had tumors in both locations. The proportion of tumor stages was 17.8% for pTa/pTis, 23.7% for pT1, 21.6% for pT2, 30.3% for pT3, and 6.6% for pT4. Lymph node metastasis was found in 6.8% of patients. In terms of tumor grade, low grade and high grade were 129 cases (17.7%) and 601 cases (82.3%), respectively. Most patients (73.3%) only had solitary tumors in the upper urinary tract, while 195 patients (26.7%) had multiple tumors. In all, 10 (1.3%) and 126 (17.3%) patients received neoadjuvant and adjuvant chemotherapy, respectively.

### 3.2. Clinicopathological Features in High and Low HRR Groups

In Table 1, 730 patients were divided into two groups, 603 cases in the low HRR (≤1.05) group and 127 cases in the high HRR (>1.05) group. The analysis showed that there were no differences in the history of bladder cancer, tumor location, nodal status, focality, and WBC count between the two groups. However, patients in the high HRR group were significantly younger (63.58 ± 10.21 vs. 68.15 ± 10.37, *p* < 0.00001), predominantly male (*p* < 0.00001), and had higher hemoglobin levels (14.45 ± 0.91 vs. 11.11 ± 1.69, *p* < 0.00001), lower RDW (12.72 ± 0.59 vs. 14.17 ± 2.14, *p* < 0.00001), lower chronic kidney disease stage (*p* < 0.00001), more low-grade tumors (*p* = 0.028), a greater chance of receiving laparoscopic surgery (*p* = 0.016), milder pathological stage (*p* = 0.009), and lower platelet count (*p* = 0.002).

### 3.3. Kaplan–Meier Analysis

**Figure 1:**
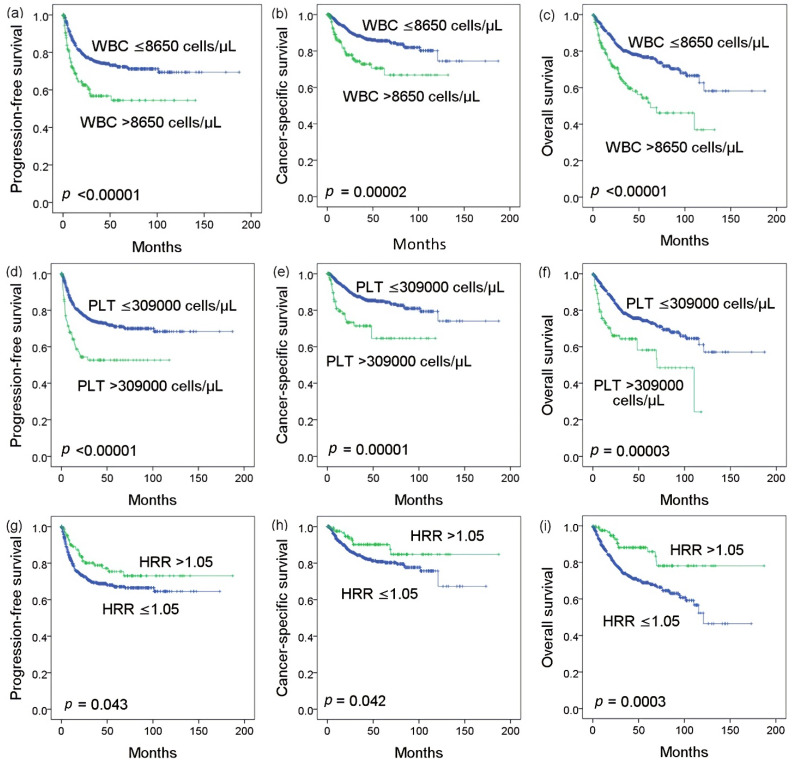
Kaplan–Meier analysis showing that progression-free survival, cancer-specific survival, and overall survival are significantly associated with white blood cell (WBC) count (**a**–**c**), platelet (PLT) count (**d**–**f**), and hemoglobin-to-red cell distribution width ratio (HRR) level (**g**–**i**).

Figure 1 shows the Kaplan–Meier curve of PFS, CSS, and OS according to WBC count, platelet count, and HRR level. The mean follow-up was 41.74 months. We found that patients with elevated WBC or platelet counts had poor PFS (Figure 1a,d, both *p* < 0.00001), CSS (Figure 1b,e, both *p* < 0.0001) and OS (Figure 1c,f, both *p* < 0.0001). The 5-year PFS, CSS and OS of patients with low HRR were also significantly worse (Figure 1g: 67.1% vs. 75.4%, *p* = 0.043; Figure 1h: 80.8% vs. 90.2%, *p* = 0.042; Figure 1i: 68.9% vs. 85.8%, *p* = 0.0003, respectively).

### 3.4. Cox Regression Analysis

Table 2 lists the results of univariate analysis for PFS, CSS, and OS. Older age was associated with inferior OS (*p* = 0.001), but not related to PFS and CSS. In terms of gender, male patients had significantly worse PFS (*p* = 0.036) and CSS (*p* = 0.046). Patients with synchronous pelvicalyceal and ureteral tumors had poor prognosis (*p* = 0.0001, 0.006, and 0.001 for PFS, CSS, and OS, respectively). For pathological features, advanced pathological T stage (all *p* < 0.00001), positive lymph node involvement (all *p* < 0.00001), high tumor grade (*p* < 0.00001 for PFS, *p* = 0.0002 for CSS, and *p* = 0.00007 for OS), and multifocality (*p* = 0.00004 for PFS, *p* = 0.002 for CSS, and *p* = 0.007 for OS) were all strongly associated with poor disease outcomes. As for hemogram, high WBC count (>8.65 × 10^3^ cells/μL) and high platelet count (>309 × 10^3^ cells/μL) were related to worse PFS (both *p* < 0.00001), CSS (both *p* = 0.00003), and OS (*p* < 0.00001 and *p* = 0.00005, respectively). However, low hemoglobin (≤11.4 g/dL) was only associated with inferior OS (*p* = 0.006), and high RDW (>14.1%) was significantly related to poor PFS (*p* = 0.027) and OS (*p* < 0.00001) but not to CSS. Of note, low HRR (≤1.05) was significantly associated with dismal PFS (HR 1.529; 95% CI: 1.011–2.313), CSS (HR 1.853; 95% CI: 1.014–3.386), and OS (HR 2.493; 95% CI: 1.491–4.168).

Multivariate analyses of PFS, CSS, and OS are shown in Table 3. Older age was still associated with a significant decrease in OS (*p* = 0.015). Advanced pathological T stage (all *p* < 0.00001) and positive nodal status (*p* < 0.00001 for PFS, *p* = 0.00006 for CSS, and *p* < 0.00001 for OS) were both significant risk factors. Regarding blood cell markers, high WBC count was associated with poor prognosis (*p* < 0.00001 for PFS, *p* = 0.0001 for CSS, and *p* < 0.00001 for OS). In addition, low HRR was an independent prognostic factor for worse PFS (HR 1.873; 95% CI:1.191–2.944), CSS (HR 2.174; 95% CI:1.130–4.182), and OS (HR 2.515; 95% CI:1.460–4.331).

## 4. Discussion

HRR data are commonly available and can be widely used as a clinically applicable indicator. In our study, patients with low HRR have higher pathological stages, higher tumor grades, and worse PFS, CSS, and OS after adjusting for a variety of confounders, suggesting its correlation with advanced pathological characteristics and its value in predicting survival of UTUC patients. Therefore, it is promising to combine HRR with other important clinicopathological factors to help surgeons identify suitable candidates for multimodal treatment. However, the use of HRR in patients receiving red blood cell transfusions may be limited because transfusions can weaken its prognostic value by altering hemoglobin and RDW [29].

In multivariate analysis, we found that WBC count >8.65 × 10^3^ cells/μL was significantly associated with poor prognosis, which was similar to previous reports on UTUC [5,8,14,16]. Although each report set a different threshold, ranging from 7.6 × 10^3^ to 8.3 × 10^3^ cells/μL, they still came to the same conclusion that elevated WBC count was related to poor UTUC survival. The prognostic potential of the WBC count can be explained by the mechanism of cancer-related inflammation, which has two pathways, namely extrinsic and intrinsic pathways. Both pathways can activate transcription factors, including nuclear factor-κB and signal transducer and activator of transcription 3 (STAT3). In turn, chemokines will be produced, including interleukin 8 and CC-chemokine ligand 2 (CCL2). The cascade may induce leukocyte recruitment and angiogenesis, ultimately leading to tumor progression [30].

The prognostic value of hemoglobin has been examined in UTUC, and its possible mechanism involves hypoxia-inducible factors (HIFs), which regulate the expression of genes that contribute to angiogenesis, motility, invasion, and metastasis [31]. However, there are some inconsistencies in different studies. Among the 11 reports we reviewed [9,11,12,13,14,15,16,17,19,20,32], five of them described the association between low hemoglobin level and worsening of PFS or recurrence-free survival [9,11,14,20,32], whereas the other three cohort studies failed to demonstrate its significance [12,13,15]. Six studies concluded that low hemoglobin levels were associated with poor CSS [9,11,12,14,20,32], while Sakano et al. [16], Takahara et al. [19] and Chung et al. [11] denied this finding. Two retrospective studies showed that patients with anemia had inferior OS [11,32], but Rink et al. [17] and Qin et al. [15] could not confirm this correlation. Taken together, hemoglobin may be a useful marker, but its prognostic effect in UTUC is not consistent. In our univariate analysis, hemoglobin ≤11.4 g/dL can predict OS but not PFS or CSS.

RDW is an index used to measure the size distribution of red blood cells, which helps distinguish different types of anemia. Recently, its prognostic potential in malignant tumors has been described. Although the exact mechanism is uncertain, various hypotheses have been proposed, such as impaired functional status [33] and malnutrition [34]. Six meta-analyses reported the relationship between RDW and cancer survival [35,36,37,38,39,40]. Five of them revealed that patients with high RDW tended to have lower OS and PFS [35,36,37,38,39]. Despite this, of the three articles which analyzed the association between RDW and CSS, only one systemic review showed a significant correlation [36,39,40]. For UTUC, it was also observed that high RDW (≥14.0%) could reflect the patient’s OS but not CSS [10]. Similar to most previous meta-analyses, our study found that RDW >14.1% was associated with PFS and OS, but not with CSS. In short, RDW is a prognostic factor for OS, but its role in predicting CSS may be limited.

Although many researchers have emphasized the individual impact of hemoglobin and RDW, few people understand the relationship between HRR and prognosis in cancer patients. In this study, HRR can retain the advantages of hemoglobin and RDW, and is significantly correlated with PFS, CSS, and OS of UTUC. It is known that both hemoglobin and RDW are affected by factors that lead to tumor development or poor prognosis, such as inflammation [25,26], oxidative stress [23,24], impaired functional status [33,41], aging [42,43], malnutrition [34,44], and decreased renal function [44,45]. Since these factors can cause anemia and increase RDW, dividing hemoglobin by RDW may provide a more powerful marker than using a single parameter alone.

The underlying mechanism between HRR and carcinogenesis is unclear, and we attempt to propose the potential hypotheses. Although anemia itself causes an increase in RDW [46], their interaction with cancer is much more complicated. Inflammatory cytokines secreted by tumors, such as IL-6 and TNF-α [47,48], can change the tumor and hematopoietic microenvironment, leading to worsening tumor oxygenation [49] and tumor-related anemia [50,51]. Subsequently, hypoxia [52] and TNF-α [53] further shorten the lifespan of erythrocytes and give rise to the release of a large number of immature red blood cells in the circulation, which leads to a rise in RDW. Oxidative stress [54] and inducible cytokines, caused by hypoxia-induced HIF-1α [55] and anemia of inflammation [53], respectively, can also reduce erythrocyte survival and increase RDW [26]. From another perspective, RDW is considered to be a marker of cancer-associated inflammation [25], which can result in a decrease in red blood cell production [56]. As a common poor prognostic factor in cancer, aging is related to both hemoglobin and RDW. With age, anemia becomes more prevalent due to poor hematopoietic function or weakened response to erythropoietin [42]. RDW is involved in the aging pathways [57], and elevated RDW is also a significant indicator of cell senescence, that is, telomere shortening [43]. In summary, compared with individual components, HRR enhances the impact on carcinogenesis and represents a more effective marker.

Limited articles have investigated the effect of HRR on cancers. A study of 85 gastric cancer cases showed that patients with high HRR (≥0.89) had better PFS and OS [58]. In a retrospective study of 205 patients with head and neck cancer, low HRR (≤1.019) had a negative impact on event-free survival [59]. Another study recruited 153 patients diagnosed with non-small cell lung cancer, and the results revealed that HRR <0.88 reduced PFS and OS [60]. With the HRR cutoff value of 0.985, a similar association was also found in a report of 146 small cell lung cancer patients [61]. Yilmaz et al. showed that HRR could predict PFS and OS in 152 patients with muscle-invasive bladder cancer, and that HRR <0.94 was associated with advanced tumor stage and grade and more lymphovascular invasion and perineural invasion [62]. Consistent with previous studies, our findings also support that UTUC patients with low HRR have adverse pathological features and poor outcomes.

Our study had limitations. First of all, this was a retrospective study of a medical center and two metropolitan hospitals. Second, all the included cases were Asians, so the results needed to be verified in other ethnicities. Third, we did not collect patient nutritional data, such as serum iron, ferritin, vitamin B12, and folic acid, so these potential confounding factors could not be corrected. Finally, this study did not investigate markers derived from WBC differentials. For one reason, there was no routine check of differential counts in our practice. More importantly, analyzing these markers (such as the neutrophil to lymphocyte ratio or platelet to lymphocyte ratio) along with WBC or platelet counts in the Cox regression model was very likely to cause statistical errors due to overadjustment and collinearity. For the same reason, hemoglobin and RDW were not included in multivariate analysis.

## 5. Conclusions

Our study found that low HRR and high WBC count were independent indicators for predicting UTUC patients with poor PFS, CSS, and OS. The mechanism between HRR and carcinogenesis is complex, and the intertwined relationship may stem from tumor-related anemia, hypoxia, oxidative stress, cancer-associated inflammation, or the involvement of aging pathways. These markers are characterized by low cost and high accessibility and can serve as useful prognostic factors for UTUC. With the help of blood cell markers obtained by preoperative examination, we expect to refine our treatment strategy and follow-up program to achieve ideal personalized management. Future prospective studies are warranted to validate their clinical significance.

## Figures and Tables

**Table 1:** Demographic data of the patients.

Variables	All Patients		HRR ≤ 1.05		HRR > 1.05		*p* Value
	(*N* = 730)		(*N* = 603)		(*N* = 127)		
	*N*	%	*N*	%	*N*	%	
Age (Mean ± SD), years	67.36 ± 10.48		68.15 ± 10.37		63.58 ± 10.21		<0.00001 **
Hemoglobin (g/dL)	11.69 ± 2.03		11.11 ± 1.69		14.45 ± 0.91		<0.00001 **
RDW (%)	13.92 ± 2.04		14.17 ± 2.14		12.72 ± 0.59		<0.00001 **
Gender							<0.00001 **
Female	407	55.8	370	61.4	37	29.1	
Male	323	44.2	233	38.6	90	70.9	
Smoking							<0.00001 **
No	577	79	499	82.8	78	61.4	
Yes	153	21	104	17.2	49	38.6	
Chronic kidney disease							<0.00001 **
Stage 1 (≥90 mL/min/1.73 m^2^)	61	8.4	44	7.3	17	13.4	
Stage 2 (60–89 mL/min/1.73 m^2^)	195	26.7	135	22.4	60	47.2	
Stage 3 (30–59 min/1.73 m^2^)	294	40.3	245	40.6	49	38.6	
Stage 4 (15–30 mL/min/1.73 m^2^)	57	7.8	57	9.5	0	0	
Stage 5 (<15 mL/min/1.73 m^2^)	123	16.8	122	20.2	1	0.8	
History of bladder cancer							0.241
No	619	84.8	507	84.1	112	88.2	
Yes	111	15.2	96	15.9	15	11.8	
Tumor location							0.68
Renal pelvis	319	43.7	262	43.4	57	44.9	
Ureter	270	37	221	36.7	49	38.6	
Synchronous	141	19.3	120	19.9	21	16.5	
Approach							0.016 *
Open	358	49	308	51.1	50	39.4	
Laparoscopy	372	51	295	48.9	77	60.6	
Pathological T stage							0.009 **
pTa/pTis	130	17.8	108	17.9	22	17.3	
pT1	173	23.7	137	22.7	36	28.3	
pT2	158	21.6	134	22.2	24	18.9	
pT3	221	30.3	176	29.2	45	35.4	
pT4	48	6.6	48	8	0	0	
Nodal status							0.252
pN0	188	25.8	158	26.2	30	23.6	
pNx	492	67.4	400	66.3	92	72.4	
pN1/pN2	50	6.8	45	7.5	5	3.9	
Grade							0.028 *
Low	129	17.7	98	16.3	31	24.4	
High	601	82.3	505	83.7	96	75.6	
Focality							0.519
Unifocal	535	73.3	439	72.8	96	75.6	
Multifocal	195	26.7	164	27.2	31	24.4	
WBC							0.662
≤8.65 × 10^3^ cells/μL	564	77.3	464	76.9	100	78.7	
>8.65 × 10^3^ cells/μL	166	22.7	139	23.1	27	21.3	
Platelet							0.002 **
≤309 × 10^3^ cells/μL	634	86.8	513	85.1	121	95.3	
>309 × 10^3^ cells/μL	96	13.2	90	14.9	6	4.7	

HRR hemoglobin-to-red cell distribution width ratio, RDW red blood cell distribution width, WBC white blood cell, SD standard deviation, * < 0.05, ** < 0.01.

**Table 2:** Comparative univariate survival analyses of 730 patients with upper tract urothelial carcinoma (UTUC).

Univariate Analysis	Progression-Free Survival		Cancer-Specific Survival		Overall Survival	
	HR (95% CI)	*p* Value	HR (95% CI)	*p* Value	HR (95% CI)	*p* Value
Age, years	1.009 (0.995, 1.023)	0.198	1.016 (0.996, 1.035)	0.112	1.026 (1.011, 1.041)	0.001 **
Gender		0.036 *		0.046 *		0.473
Female	1		1		1	
Male	1.354 (1.019, 1.798)		1.484 (1.008, 2.187)		1.114 (0.830, 1.496)	
Smoking		0.092		0.082		0.082
No	1		1		1	
Yes	1.327 (0.955, 1.845)		1.478 (0.952, 2.294)		1.353 (0.962, 1.903)	
Chronic kidney disease		0.015		0.149		0.325
Stage 1 (≥90 mL/min/1.73 m^2^)	1		1		1	
Stage 2 (60–89 mL/min/1.73 m^2^)	2.128 (1.056, 4.288)	0.035	1.285 (0.561, 2.944)	0.552	0.730 (0.425, 1.253)	0.253
Stage 3 (30–59 mL/min/1.73 m^2^)	2.159 (1.088, 4.283)	0.028	1.556 (0.706, 3.428)	0.273	0.901 (0.546, 1.486)	0.682
Stage 4 (15–30 mL/min/1.73 m^2^)	1.629 (0.686, 3.867)	0.269	1.515 (0.549, 4.182)	0.422	1.003 (0.503, 2.003)	0.992
Stage 5 (<15 mL/min/1.73 m^2^)	1.091 (0.497, 2.396)	0.829	0.642 (0.239, 1.724)	0.379	0.607 (0.331, 1.115)	0.108
History of bladder cancer		0.4		0.136		0.568
No	1		1		1	
Yes	1.177 (0.805, 1.721)		1.450 (0.890, 2.364)		1.125 (0.751, 1.684)	
Tumor location		0.0002 **		0.015 *		0.005 **
Renal pelvis	1		1		1	
Ureter	1.042 (0.745, 1.457)	0.812	1.118 (0.709, 1.764)	0.632	1.303 (0.927, 1.831)	0.128
Synchronous	1.993 (1.401, 2.834)	0.0001 **	1.975 (1.218, 3.203)	0.006 **	1.887 (1.290, 2.761)	0.001 *
Approach		0.164		0.013 *		0.020 *
Open	1		1		1	
Laparoscopy	0.817 (0.614, 1.086)		0.605 (0.406, 0.900)		0.702 (0.522, 0.946)	
Pathological T stage		<0.00001 **		<0.00001 **		<0.00001 **
pTa/pTis	1		1		1	
pT1	3.277 (1.103, 9.739)	0.033 *	4.594 (0.553, 38.165)	0.158	1.089 (0.550, 2.156)	0.807
pT2	8.439 (3.008, 23.678)	0.00005 **	14.171 (1.879, 106.879)	0.010 **	2.149 (1.153, 4.006)	0.016 *
pT3-4	24.907 (9.205, 67.397)	<0.00001 **	56.131 (7.806, 403.618)	0.000006 **	5.596 (3.205, 9.768)	<0.00001 **
Nodal status		<0.00001 **		<0.00001 **		<0.00001 **
pN0	1		1		1	
pNx	0.827 (0.590, 1.160)	0.271	0.814 (0.510, 1.298)	0.387	0.830 (0.586, 1.174)	0.292
pN1/pN2	5.197 (3.341, 8.086)	<0.00001 **	7.018 (3.934, 12.518)	<0.00001 **	5.714 (3.613, 9.036)	<0.00001 **
Grade		<0.00001 **		0.0002 **		0.00007 **
Low	1		1		1	
High	4.431 (2.409, 8.149)		9.148 (2.898, 28.879)		2.833 (1.693, 4.740)	
Focality		0.00004 **		0.002 **		0.007 **
Unifocal	1		1		1	
Multifocal	1.855 (1.383, 2.489)		1.880 (1.265, 2.795)		1.530 (1.123, 2.084)	
WBC		<0.00001 **		0.00003 **		<0.00001 **
≤8.65 × 10^3^ cells/μL	1		1		1	
>8.65 × 10^3^ cells/μL	2.026 (1.489, 2.758)		2.400 (1.592, 3.618)		2.264 (1.652, 3.104)	
Platelet		<0.00001 **		0.00003 **		0.00005 **
≤309 × 10^3^ cells/μL	1		1		1	
>309 × 10^3^ cells/μL	2.230 (1.573, 3.161)		2.673 (1.690, 4.227)		2.154 (1.487, 3.120)	
Hemoglobin		0.752		0.077		0.006 *
>11.4 g/dL	1		1		1	
≤11.4 g/dL	1.047 (0.787, 1.394)		1.417 (0.963, 2.086)		1.506 (1.123, 2.019)	
RDW		0.027 *		0.06		<0.00001 **
≤14.1%	1		1		1	
>14.1%	1.401 (1.039, 1.888)		1.471 (0.983, 2.201)		1.988 (1.478, 2.673)	
HRR		0.044 *		0.045 *		0.0005 **
>1.05	1		1		1	
≤1.05	1.529 (1.011, 2.313)		1.853 (1.014, 3.386)		2.493 (1.491, 4.168)	

WBC white blood cell, RDW red blood cell distribution width, HRR hemoglobin-to-red cell distribution width ratio, HR hazard ratio, CI confidence interval, * < 0.05, ** < 0.01.

**Table 3:** Comparative multivariate survival analyses of 730 patients with upper tract urothelial carcinoma (UTUC).

Multivariate Analysis	Progression-Free Survival		Cancer-Specific Survival		Overall Survival	
	HR (95% CI)	*p* Value	HR (95% CI)	*p* Value	HR (95% CI)	*p* Value
Age, years					1.019 (1.004, 1.034)	0.015 **
Gender		0.149		0.063		
Female	1		1			
Male	1.247 (0.924, 1.682)		1.472 (0.980, 2.213)			
Chronic kidney disease		0.122				
Stage 1 (≥90 mL/min/1.73 m^2^)	1					
Stage 2 (60–89 mL/min/1.73 m^2^)	1.319 (0.643, 2.706)	0.45				
Stage 3 (30–59 mL/min/1.73 m^2^)	1.086 (0.534, 2.210)	0.82				
Stage 4 (15–30 mL/min/1.73 m^2^)	0.711 (0.292, 1.733)	0.453				
Stage 5 (<15 mL/min/1.73 m^2^)	0.713 (0.320, 1.592)	0.41				
Tumor location		0.223		0.657		0.094
Renal pelvis	1		1		1	
Ureter	0.994 (0.695, 1.422)	0.975	1.047 (0.646, 1.699)	0.852	1.265 (0.888, 1.803)	0.193
Synchronous	1.553 (0.904, 2.666)	0.111	1.396 (0.673, 2.893)	0.37	1.823 (1.043, 3.187)	0.035*
Approach				0.324		0.559
Open			1		1	
Laparoscopy			0.809 (0.531, 1.233)		0.911 (0.666, 1.246)	
Pathological T stage		<0.00001 **		<0.00001 **		<0.00001 **
pTis/pTa	1		1		1	
pT1	3.057 (1.007, 9.279)	0.049 *	4.031 (0.473, 34.334)	0.202	1.110 (0.549, 2.244)	0.772
pT2	7.761 (2.696, 22.339)	0.0001 **	10.930 (1.394, 85.669)	0.023 *	1.872 (0.959, 3.656)	0.066
pT3-4	16.961 (5.933, 48.486)	<0.0001 **	32.687 (4.274, 249.986)	0.0008 **	4.226 (2.227, 8.020)	0.00001 **
Nodal status		<0.00001 **		0.00006 *		<0.00001 **
pN0	1		1		1	
pNx	0.958 (0.679, 1.352)	0.807	1.021 (0.631, 1.650)	0.934	0.974 (0.682, 1.391)	0.885
pN1/pN2	2.623 (1.664, 4.136)	0.00003 **	3.207 (1.777, 5.789)	0.0001 *	3.077 (1.918, 4.934)	<0.00001 **
Grade		0.27		0.216		0.418
Low	1		1		1	
High	1.448 (0.750, 2.794)		2.145 (0.640, 7.187)		1.274 (0.708, 2.292)	
Focality		0.262		0.501		0.709
Unifocal	1		1		1	
Multifocal	1.310 (0.817, 2.099)		1.237 (0.666, 2.300)		0.913 (0.567, 1.471)	
WBC		<0.00001 **		0.0001 **		<0.00001 **
≤8.65 × 10^3^ cells/μL	1		1		1	
>8.65 × 10^3^ cells/μL	2.212 (1.568, 3.122)		2.387 (1.513, 3.768)		2.371 (1.674, 3.358)	
Platelet		0.45		0.259		0.318
≤309 × 10^3^ cells/μL	1		1		1	
>309 × 10^3^ cells/μL	1.166 (0.783, 1.739)		1.351 (0.801, 2.280)		1.237 (0.815, 1.878)	
HRR		0.007 **		0.020 *		0.0009 **
>1.05	1		1		1	
≤1.05	1.873 (1.191, 2.944)		2.174 (1.130, 4.182)		2.515 (1.460, 4.331)	

WBC white blood cell, HRR hemoglobin-to-red cell distribution width ratio, HR hazard ratio, CI confidence interval, * < 0.05, ** < 0.01.

## Data Availability

The data presented in this study are available upon reasonable request from the corresponding author. The data are not publicly available due to patient privacy.
